# Stressful Newborn Memories: Pre-Conceptual, *In Utero*, and Postnatal Events

**DOI:** 10.3389/fpsyt.2019.00220

**Published:** 2019-04-18

**Authors:** Zoe Papadopoulou, Angeliki-Maria Vlaikou, Daniela Theodoridou, Georgios S. Markopoulos, Konstantina Tsoni, Eleni Agakidou, Vasiliki Drosou-Agakidou, Christoph W. Turck, Michaela D. Filiou, Maria Syrrou

**Affiliations:** ^1^Laboratory of Biology, Faculty of Medicine, School of Health Sciences, University of Ioannina, Ioannina, Greece; ^2^Laboratory of Biochemistry, Department of Biological Applications and Technology, School of Health Sciences, University of Ioannina, Ioannina, Greece; ^3^1^st^ Department of Neonatology and Neonatal Intensive Care Unit, Medical Faculty, Aristotle University School of Health Sciences, Thessaloniki, Greece; ^4^Max Planck Institute of Psychiatry, Munich, Germany

**Keywords:** stress, predisposition, epigenetics, low birth weight, individuality, early-life stress, mitochondria

## Abstract

Early-life stressful experiences are critical for plasticity and development, shaping adult neuroendocrine response and future health. Stress response is mediated by the autonomous nervous system and the hypothalamic–pituitary–adrenal (HPA) axis while various environmental stimuli are encoded *via *epigenetic marks. The stress response system maintains homeostasis by regulating adaptation to the environmental changes. Pre-conceptual and *in utero* stressors form the fetal epigenetic profile together with the individual genetic profile, providing the background for individual stress response, vulnerability, or resilience. Postnatal and adult stressful experiences may act as the definitive switch. This review addresses the issue of how preconceptual *in utero* and postnatal events, together with individual differences, shape future stress responses. Putative markers of early-life adverse effects such as prematurity and low birth weight are emphasized, and the epigenetic, mitochondrial, and genomic architecture regulation of such events are discussed.

## Stress, Brain, and The Environment

Physiological or biological stress is the response to a stressor, i.e., an environmental condition or a stimulus. Τhe body responds to stress by sympathetic nervous system activation as a result of the fight-or-flight response. The stress response aims to restore homeostatic control and facilitate adaptation. The brain processes stress in three main areas: amygdala, hippocampus, and prefrontal cortex (PFC). Amygdala and hippocampus play a critical role in memory formation and are associated with anxiety, fear, and cognitive processes. PFC is the brain region linked to planning complex cognitive behavior, personality expression, decision making, and moderating social behavior (1). The basic activity of the PFC region is to orchestrate thoughts and actions in accordance with internal goals and executive function (2). Corticosteroid receptors that react to the stressor through steroid hormone binding are abundant in these areas (3, 4). It is well established that stressful experiences during critical periods of early brain development can affect emotional and behavioral functions in adult life (5). The autonomous nervous system and the hypothalamic–pituitary–adrenal (HPA) axis are responsible for these functions and mediate stress response through targeted hormone release. This system acts by negative feedback to maintain brain homeostasis. The hypothalamus is stimulated by its inputs and releases the corticotropin-releasing hormone. This hormone is transported to its target, the pituitary gland, where it binds to the targeted receptors and causes the release of the adrenocorticotropic hormone. Although the main purpose of this system is well understood, recent studies attempt to identify underlying genetic mechanisms of brain function modulating mediators of this system including adrenaline and neuropeptides (6). Glucocorticoids reach the brain through the peripheral blood flow, where they bind to specific types of cytoplasmic glucocorticoid receptors (GRs) and mineralocorticoid receptors (MRs). MRs make up the majority of stress corticosteroid receptors with a high affinity for cortisol and are activated as soon as a stressor appears. GRs have a low affinity for cortisol and are only activated when stress reaches its peak on the brain. This complex is then translocated to the nucleus, where it binds to specific DNA elements [glucocorticoid response elements (GREs)] and acts as a transcription factor activating or repressing a great number of genes (7).

## Early-Life Stress, Learning, and Memory

Exposure to early-life stressful events has been shown to activate the HPA stress hormone system. HPA axis mediator and receptor genes are prime targets of epigenetic modifications by DNA methylation and histone acetylation (8). The combination of genetic and epigenetic factors affects cell function and brain development. As a result, individuals who have experienced chronic stress during early development and childhood are at high risk for a wide range of behavioral problems that persist into adulthood. This phenotype becomes evident by learning and emotion regulation difficulties, alcohol and substance abuse, externalizing problems, as well as depression and anxiety disorders (7). Children who have experienced maltreatment or were exposed to maternal deprivation trauma have shown poor performance in tasks involving working memory, attention, planning, and learning processes (9, 10). In rodents, maternal deprivation is a well-established paradigm of early-life stress. Maternal deprivation of newborns from their dam leads to epigenetic changes in specific imprinted genes and dysfunctions. Behavioral and molecular effects depend on the duration and type of maternal deprivation and individual predisposition (11).

## *In Utero* Stress Exposures

Intrauterine life events may have a much greater impact on epigenetic profiles than stressful exposures during adult life (12). Early stages of embryonic development are characterized by heightened brain plasticity that is adversely affected by exposure to environmental insults (13). Complex gene environment interactions during critical early developmental periods may have lasting effects and result in adult psychopathology (14, 15). Maternal stress exposure, anxiety, and depression during pregnancy are considered *in utero* adverse experiences and have been associated with low birth weight (LBW) and future health problems (16–24). LBW, apart from being a risk factor for neonatal morbidity and mortality, has been proposed as a marker of early-life adversities (25, 26).

In this mini-review, genetic and epigenetic factors that shape stress response are discussed. The contribution of mitochondria and individual predisposition to developing mental health problems in response to a stressful stimulus will also be addressed.

## Genetics and Epigenetics of The Stress Response

**Figure 1:**
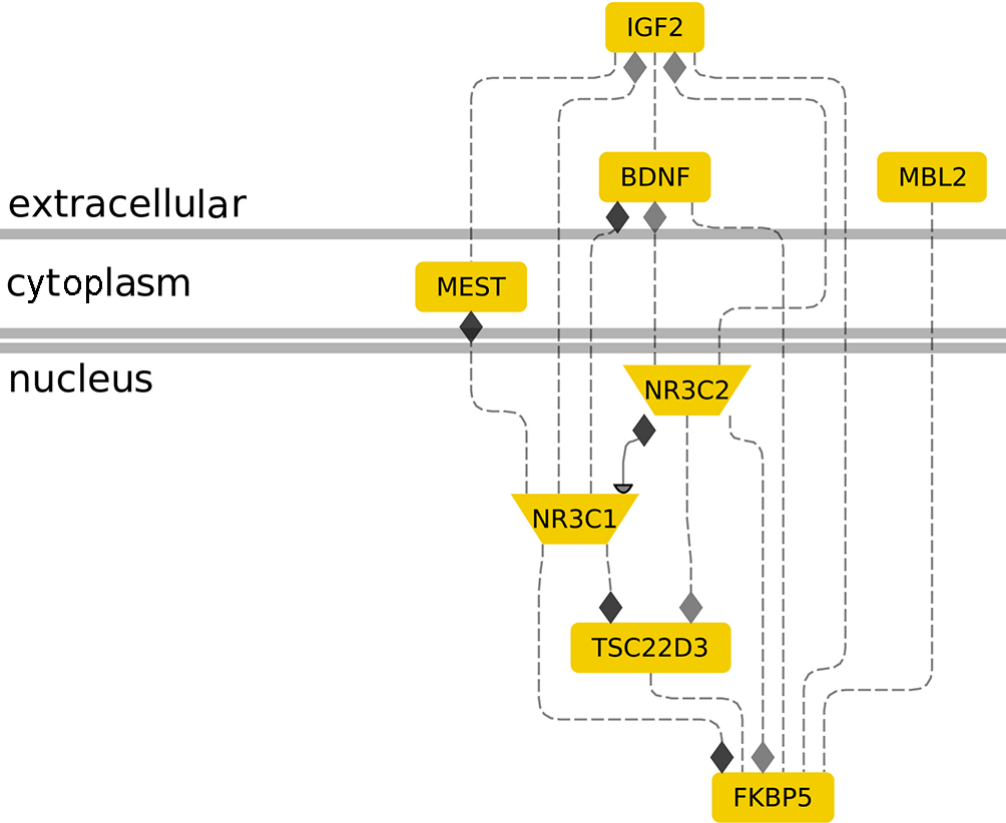
The role of molecular genetic markers in cellular stress response. The interconnection between genes *IGF2, MBL2, MEST, NR3C1, NR3C2, TSC22D3*, and *BDNF* in the context of stress response is shown. Membrane-bound *IGF2* induction by maternal distress is associated with nuclear *NR3C2* induction (3). *IGF2* shows similar changes in methylation levels with NR3C1 during age-related stress (4), *FKBP5* during the development of preterm infants (30), and *MEST* in infertile males (31). *MBL2* and *FKBP5* upregulation has been associated with parental-nutrition-induced stress (32, 33). NR3C2 and *NR3C1* interact to control gene expression during stress (8, 34). Nuclear *NR3C1* (glucocorticoid receptor) seems to be a convergence point for *FKBP5* (9, 35), *NR3C2* (10, 36), *MEST* (11, 37), and *IGF2* (4) action in stress. *TSC22D3* is an established glucocorticoid signaling responsive gene that is regulated by *NR3C1* (27, 38) and an *NR3C2* target (28). *BDNF* is upregulated by *IGF2* (39) in an Alzheimer’s disease mouse model, inhibited by *NR3C1* (40) in neuron-like cells, and associated with high *NR3C2* and low *NR3C1* in high-cholesterol-diet rats (41). *FKBP5* elevation is associated with *BDNF* suppression and improved anxiety, depression, and posttraumatic stress disorder conditions (42). Gene interaction analysis was performed using the Genomatix Pathway System (Genomatix.de).

Early-life adversities have been implicated in the occurrence of neuropsychiatric conditions, such as, Post-traumatic stress disorder (PTSD), depression, psychosis, and phenotypes resembling mood- and anxiety-related disorders (4–8). Recent data are beginning to unravel the complex interactions between genes and environment, namely, an individual’s genetic and epigenetic profile that renders the person resilient or at risk for developing a stress-related disorder (9, 10). Apart from the genetics of neuroendocrine stress response, it is important to take into consideration its epigenetic profile (11). A plethora of epigenetic marks, contributing to either the enhanced or suppressed expression of a gene, in combination with risk- or resilience-related predisposing polymorphisms, shape an individual’s phenotype (27). The complex interaction of the genetic background with the epigenetic profile that reflects early-life experiences and is potentially reversible by environmental factors can result in a phenotype that is either resilient or sensitive towards adverse stress exposures (28, 29). Several genes and their epigenetic regulation have been implicated in the susceptibility to early-life stress. An overview of the below-discussed genes and their interrelations is provided in **Figure 1**.

*NR3C1* and *NR3C2* genes (Nuclear Receptor Subfamily 3 Group C Member 1 and 2), encoding the GR and MR, respectively, are widely expressed in limbic regions of the brain and regulate HPA axis activity by cortisol binding. Deregulation of the GR–MR function may lead to HPA axis malfunction and stress vulnerability (43–45). The *NR3C1* gene, localized on the 5q31-32 chromosome, contains nine exons (1–9) (45, 46). In the 5′ Untranslated region (UTR), alternative splice variants of the first exon form the distal and proximal gene promoter that contains a crucial CpG island regulating the expression of exon 1_F_. The multiple alternative first exon splice variants render the expression of *NR3C1* tissue-specific (47–50). The first study in humans examining the epigenetic status of 1_F_ promoter in low prenatal and increased maternal postnatal depression showed elevated methylation levels. This effect is reversed by maternal stroking of the newborns during the first postnatal weeks (51). In a thorough meta-analysis, psychosocial maternal prenatal stress was significantly correlated with DNA methylation at CpG 36 of the 1_F_ promoter (52). Interestingly, prenatal exposure of depressed mothers to serotonin reuptake inhibitors was not associated with alterations in the methylation profile of the 1_F _promoter. However, a correlation between the psychological profiles of depressed mothers, especially during the third trimester, and increased HPA axis reactivity of the newborns, has been reported (53). Maternal anxiety during the first two trimesters also affects the methylation status of *NR3C1*, thus diminishing *NR3C1* gene expression (54). In a study examining the effects of maternal-related stressors such as maternal deprivation due to financial difficulties, daily psychosocial stress, and war-related phenomena, a strong correlation was found between the aforementioned maternal stressors, neonatal birth weight, and methylation of multiple CpG sites in the upstream *NR3C1 *promoter. These results support the hypothesis that intrauterine development and maternal environmental stressors affect the plasticity and adaptation to adverse stimuli (55). Further supporting this notion, decreased expression of *NR3C1 *was observed in hippocampal tissues of suicide completers abused during childhood. These findings can be explained by alterations in hippocampal methylation of tissue-specific *NR3C1*, which persist into adulthood and lead to changes in HPA axis function (56–58). The *NR3C2* gene on 4q31.1 has recently been associated with behavioral abnormalities. Cognitive ability following acute stress has been associated with genetic variation of the GR–MR. Specifically, single-nucleotide polymorphisms (SNPs) of the above genes seem to affect cognition and HPA axis function (59, 60). In individuals with a history of childhood maltreatment, the minor *NR3C2* allele rs17581262 was correlated, among others, with lower amygdala and hippocampal volumes and major depression, suggesting that this allele is a predisposing risk factor for stress-related disorders (61).

*FKBP5* (6p21.31) encodes a 51-kDa immunophilin, which is a major component of the GR heterocomplex. Upon stress exposure, cortisol diffuses into the cytoplasm and binds the GR (62–64). *FKBP5* slows down the translocation of GR to the nucleus (65, 66). *FKBP5* expression is regulated by GREs *via* a cortisol-dependent short negative feedback loop (67, 68). Ιn intron 2 of *FKBP5* and close to a functional GRE, the significant SNP rs1360780 was identified (69). Structurally, the rare risk allele alters the chromatin conformation after GR binding to the GRE, inducing the transcription of *FKBP5*. In the presence of the protective allele, this induction is absent (67, 69). The aforementioned SNP has been linked to a variety of mental health conditions including depression, anxiety, psychosis, and posttraumatic stress disorder (70–72). During their *in utero* formation, brain regions including the amygdala and hippocampus are particularly vulnerable in cases of antenatal maternal depression and anxiety (73, 74). *FKBP5* genetic variation among neonates combined with antenatal maternal depression can predispose toward the development of depressive symptoms in the offspring later in life due to alterations in neonatal brain regions (75). Interestingly, recent reports on the association of depression with childhood maltreatment did not report FKBP5 methylation to be involved in mediatory mechanisms (76, 77).

Alterations in GR function through *NR3C1* lead to a rare endocrinological condition known as Primary Generalized Glucocorticoid Resistance (PGGR, Chrousos syndrome) (78, 79). Mutations in the *NR3C1* gene result in receptor conformation changes and low ligand binding affinity and contribute to the clinical profile and pathogenesis (80–83). PGGR is characterized by decreased tissue sensitivity toward cortisol, resulting in malfunctioning negative feedback loops (84, 85). This causes a compensatory activation of the HPA axis and hypersecretion of its end products (80, 85, 86). Interestingly, *FKBP5* has been implicated in glucocorticoid resistance. The gene’s overexpression is considered to be responsible for the low ligand-binding affinity of the GR in New World primates, providing a selective advantage of an overall normal adrenal function but with high concentrations of circulating Adrenocorticotropic hormone (ACTH) and cortisol (87, 88).

Brain-derived neurotrophic factor (BDNF) is a neurotrophin expressed in hippocampus and PFC affecting neuron survival, development, and plasticity. Early-life stress and Val66Met polymorphism result in lower BDNF availability (29, 89).

Τhe *GILZ* (glucocorticoid-induced leucine zipper) or *TSC22D3* gene, located on Xq22.2 (90), is induced by cortisol-bound GR. This complex binds on the GRE in the promoter of *GILZ*, thus rendering this gene a valid measure of GR function (91–93). In an avian species, *GILZ* expression in the pituitary gland seems to be upregulated by glucocorticoids during the second half of the embryonic development and possibly plays a role in regulating pituitary hormone expression levels (94). *GILZ *is widely expressed in the brain, and its function depends on HPA axis activation. Increased expression of *GILZ *was found in the hippocampus and medial PFC of stressed mice, indicating a region-specific function (95). In human studies, decreased *GILZ* Messenger RNA (mRNA) levels were found in the PFC and the amygdaloid nuclei in teenage suicide completers (96). The above findings are only beginning to decipher the role of *GILZ* both in stress regulation and in immune system function.

## Genetics and Epigenetics of Early Embryonic Development

*MBL2* (mannose binding lectin 2) is an important regulator of innate immunity and inflammatory processes. The *MBL2* gene encodes for a protein that assembles into a mannose-binding lectin complex. *MBL2* plays a very important role in the first-line immune responses, as a component of neonate immunity when the adaptive immunity system is not sufficiently developed (97). In humans, *MBL2* expression levels are determined genetically by a number of polymorphic sites of the gene as well as in its promoter region. Three non-synonymous SNPs, which are linked to absence or low levels of *MBL2*, have been identified in exon 1 and the promoter region. The most important *MBL2* gene SNPs associated with early infection and preterm delivery risk are variants B [rs1800450 (GGC→GAC)], C [rs1800451 (GGA→GAA)], and D [rs5030737 (CGT→TGT)]. Moreover, there are SNPs in the promoter region at position −550 in variant H/L (rs11003125) and at position −221 in variant X/Y (rs7096206) (25, 98). These *MBL2* gene polymorphisms are associated with an increased risk of perinatal and neonatal infections and risk of premature delivery (99, 100). *MBL2* levels could not predict the risk of newborn morbidity or mortality as a single factor since morbidity is also affected by other factors including sex, premature delivery, birth weight, etc. (97).

*IGF2* (insulin growth factor 2), an imprinted gene, acts as a growth factor promoting differentiation and metabolism and plays an important role in the development and nutritional needs of the fetus (101). *IGF2* and *H19* are two genes of the same imprinted domain expressed from the paternal and maternal allele, respectively, that have been implicated in the control of placental and embryonic growth through cell proliferation and apoptosis (102, 103). *H19* is crucial for growth and differentiation of the placenta (104, 105).

*MEST* (mesoderm specific transcript, 7q32) is a paternally expressed imprinted gene, which influences placental and embryonic growth, as well as birth weight of the infant (31, 106). *MEST* is a member of the a/b-hydrolase superfamily and expressed in the embryonic mesoderm (107). Increased *MEST* expression is linked to infants with high birth weight. Decreased *MEST* gene expression is observed in premature embryos compared to normal embryos, but does not affect DNA methylation (108).

## Maternal Stress and Mitochondria

Moving from single genes to subcellular functional systems, converging lines of evidence have pointed to an important role of mitochondria, the traditional “powerhouses of the cell,” as regulators of the stress response (109–111). Given the maternal origin and inheritance of mitochondria, it is plausible that maternal stress may exercise its effects on the offspring *via* alterations of mitochondrial pathways in both the *in utero* maternal microenvironment and offspring. Along these lines, it has been shown that maternal prenatal stress affects mitochondrial protein expression in pathways related to mitochondrial biogenesis and energy production in PFC and hippocampus of male rat offspring (112). Early-life maternal deprivation leads to a decrease in mitochondrial-related muscle gene expression in adult rats. Interestingly, adult-onset chronic stress had no effect on mitochondrial-related muscle gene expression function, indicating an early-life stress-specific effect (113). In humans, maternal psychosocial stress has been reported to alter the expression of mitochondrial proteins in the placenta (114). In this study, a link between mitochondrial changes and infant temperament has also been suggested. Maternal psychosocial stress and lifetime trauma have been associated with decreased mitochondrial DNA copy number in the placenta (115, 116).

## Individuality

Chronic stress links changes in the epigenetic landscape with health conditions (117). Different cell types are characterized by distinct patterns of gene expression due to developmental, environmental, physiological, and pathological reasons (117). Epigenetic mechanisms affect gene function in a dynamic way as a result of different environmental exposures during fetal development. Early-life stressful experiences, such as nutritional deprivation, lack of maternal care, or chemical exposure during critical developmental periods, can lead to phenotypic differences later in life (118). In addition to genetic susceptibility (polymorphisms, genomic architecture) inter-individual phenotypic variations are also the result of epigenetic modifications. Once we realize how different environmental triggers affect the individual epigenetic processes, we may be able to develop new means to prevent or reverse environmentally driven epigenetic changes. A recent study supports this theory and suggests that adaptation to stress is a combination of three important factors: genetic predisposition, early-life environment, and late-life environment (119). In animal models, strain, age, sex, frequency, and duration of the stressor, time point within the light cycle and temperature, and even the housing conditions are some of the environmental factors that shape the stress response(120–122). In humans, genetic background, age, sex, type, frequency, and duration of the stressor and developmental stage have been suggested to be important factors that shape individual stress response (123).

## Discussions Perspectives

**Figure 2:**
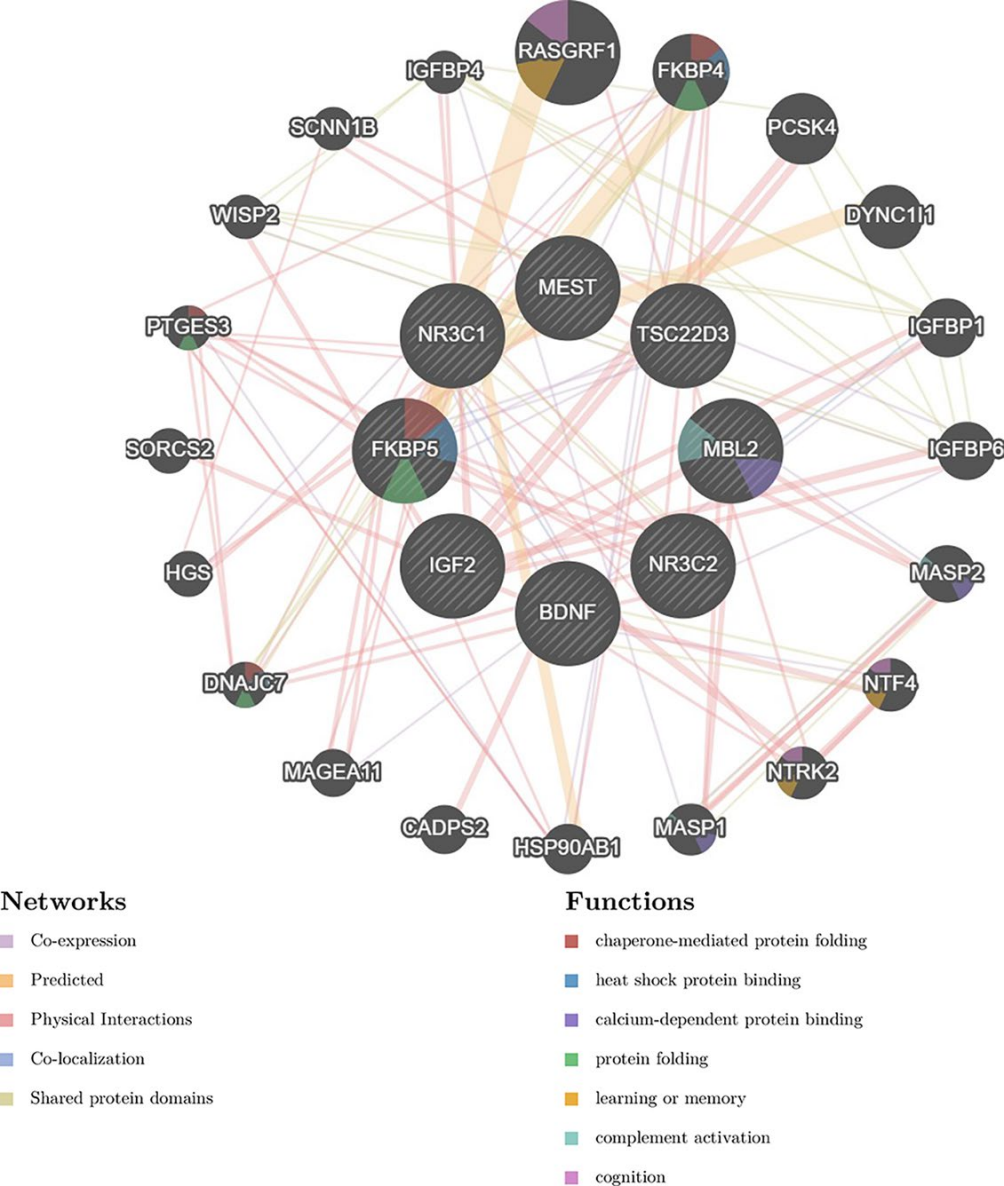
Gene interaction network discussed in the current review. The network was generated by the GeneMANIA prediction server (124). The left panel presents the different types of interactions with respective color coding, depicted with lines connecting genes in the network: physical interactions (pink), predicted (orange), co-expression (purple), and shared protein domains. The right panel presents gene functions, depicted with colored slices inside the respective genes: chaperone-mediated protein folding (red), heat shock protein binding (blue), calcium dependent protein binding (dark blue), protein folding (green), learning of memory (orange), complement activation (light blue), cognition (purple).

Early-life stress can influence brain plasticity with lasting effects. Epigenetic factors including type of exposure, timing, and diversity of experience in combination with genetic predisposition contribute to the individual resilience or vulnerability toward stress. Elucidating the interplay and downstream affected pathways (**Figure 2**) among i) housekeeping genes of the reproductive system, ii) regulators of the HPA axis, iii) components of mitochondrial heterogeneity, and iv) individual genomic architecture will facilitate our understanding of the impact of early-life stressful events for later life outcomes. Our analysis reveals the top 20 “satellite” genes (**Figure 2**) that form a functional network, affecting and being affected by the core genes controlling early-life stress. Potentially stressful or compensatory individual experiences during lifetime may have an impact on the epigenetic landscape, thus masking the effects of early-life experiences. An improved understanding will allow an integrated, systemic approach to address pathological stress responses and pinpoint novel molecular targets for pharmacological and therapeutic interventions.

## Author Contributions

All authors contributed to the writing and editing of the manuscript.

## Funding

This work has received funding from the Hellenic Foundation for Research and Innovation (HFRI) and the General Secretariat for Research and Technology (GSRT), under grant agreement No 660.

## Conflict of Interest Statement

The authors declare that the research was conducted in the absence of any commercial or financial relationships that could be construed as a potential conflict of interest.
